# Developing archetypes for key roles in a pragmatic trial: implementing human-centered design to promote advance care planning in primary care

**DOI:** 10.1186/s43058-024-00683-9

**Published:** 2025-01-06

**Authors:** Martha Abshire Saylor, Danny Scerpella, Margo Chapin, Anushka Jajodia, Amrutha J. Kadali, Jessica L. Colburn, Valerie Cotter, Jennifer L. Wolff

**Affiliations:** 1 Johns Hopkins School of Nursing, 525 N. Wolfe St, Baltimore, MD 21205P USA; 2 Johns Hopkins Bloomberg School of Public Health, Baltimore, MD USA; 3 Johns Hopkins School of Medicine, Baltimore, MD USA

**Keywords:** Advance care planning, Archetypes, Human-centered design, Methodology

## Abstract

**Background:**

Archetypes are representations of a group of people with shared behaviors, attitudes, and characteristics. The design and use of archetypes have potential application to increase partnership and support when embedding and scaling interventions but methodological approaches have not been developed.

**Objective:**

To describe the methodology of designing archetypes for use in a pragmatic trial of advance care planning in the primary care context, SHARING Choices ((NCT04819191). We present resulting archetypes representing three key roles (primary care champion, advance care planning facilitator, and patient) in our pragmatic trial.

**Methods:**

Our process for developing archetypes involved 4 steps: 1) Identify roles for archetype development, 2) Identify Shareholders and Data Sources for Archetype Development, 3) Generate unique archetypes and their distinguishing traits, and 4) Iteratively refine archetypes through exposure, scrutiny, and shareholder input. We also developed a process map to communicate our methodology.

**Results:**

We created 6 distinct archetypes for the primary care champion role, 5 archetypes for the advance care planning facilitator role and 6 archetypes for the patient role. For each archetype we described strengths, challenges, prevailing emotions, and successful approaches to collaboration (e.g., “what works for me”). Unique opportunities for synergy between archetypes (such as with facilitator and champion) and potential challenges between archetypes (such as for facilitator and patient) suggest ways to improve training and support of key roles.

**Discussion:**

Our process for creating archetypes for use in implementation research was iterative and informative in discussion of implementation with shareholders. We expect this methodology to be useful for anticipating and analyzing many aspects of implementation.

Contributions to the Literature

- We propose a methodology for designing archetypes for use in pragmatic trials to better understand implementation.
- Archetypes reveal common users in key roles in pragmatic trials including shareholders such as clinic leaders, interventionists and patients.
- Archetypes may be useful in making decisions about implementation strategies, improving reach to diverse users, staff hiring, training and dissemination.

## Background

Implementation science has long asserted the importance of engaging with key shareholders, people and groups who use and provide healthcare, to ensure the successful implementation of evidence-based initiatives in real world settings [1, 2]. Shareholder engagement can identify and strengthen partnerships among invested group members, support education and capacity, and leverage existing expertise and experience to increase uptake and adherence to the implementation of an intervention model [3, 4]. However, engaging shareholders within research can also be challenging due to diverse perspectives, experiences, competing priorities, and lack of engagement skills among research staff [4, 5]. In interactions with organizations and shareholders during pragmatic trials, representations of shareholders or users in the form of archetypes may be highly valuable to inform, evaluate and adapt implementation strategies.

The use of archetypes and ‘personas’ is a strategy for representing shareholder perspectives that has been widely deployed in product design, business strategy, and health care to describe potential users of an intervention and help teams accommodate diverse user experiences, needs, and desires. Archetypes are individual representations of a group of people with shared behaviors, attitudes, and characteristics [6, 7]. Archetypes differ from personas, which are more detailed characters with names and specific details to address characteristics identified in archetypes [6, 7]. The process of designing an archetype involves gathering information to identify commonalities and differences between members of a group and coherently organizing these attributes to characterize key attributes, strengths, and/or challenges with a focus on being detailed enough to be generalizable but suitable for different contexts or purposes. Recent literature suggests that the process of designing archetypes can encourage compassion among medical teams and stimulate engagement with the patient population [8]. This is especially important for patient care teams interacting with older adults with complex healthcare needs. Archetypes have been used in the design of dementia care environments [9], temperature guidelines in homes for older adults [10], and in digital education programs for seniors with diabetes [11].

Although archetypes are highly relevant to meeting the needs of users in real-world settings that are foundational to implementation science, little is known about applications for amplifying implementation effectiveness through use of archetypes. Archetypes can be beneficial to understand both implementation actors and targets [12]. Archetypes of implementation targets are useful for training and improving intervention delivery, and to identify need for additional implementation strategies. One study described the impact of a novel “team mapping” process that led to the development of 30 patient (implementation target) and provider (implementation actor) archetypes, and a guidebook for how these archetypes might interact in a primary care setting [13]. Another study focused only on implementation actors who were public health workers implementing strategies to reduce communicable diseases [14]. We also identified an example in which developers created a guidebook for using archetypes to improve products and processes for researchers in the translational workforce [15]. These studies touch on the conceptual application of integrating archetypes to understand implementation strategies and their effect, but less attention has been directed at describing the process of designing archetypes for various implementation actors and targets in a pragmatic trial in healthcare.

This paper describes our adapted methodology for designing archetypes for use in implementation trials to represent perspectives of implementation actors and targets for shareholders to understand varied experiences among implementation actors and targets or when it is not feasible to gather shareholders. We adapted approaches to archetype development from the engineering and human-centered design literature, specifically applying it to the context of healthcare and pragmatic trials [16, 17]. We share a case study of our experiences designing 17 archetypes representing implementation actors and targets in SHARING Choices, a large pragmatic trial of a primary care-based advance care planning (ACP) intervention. The process of developing these archetypes could be useful to other organizations seeking to identify personnel needs, make hiring decisions, adapt intervention workflows, provide training and interpret process outcomes.

## Methodology and case study application

**Fig. 1:**
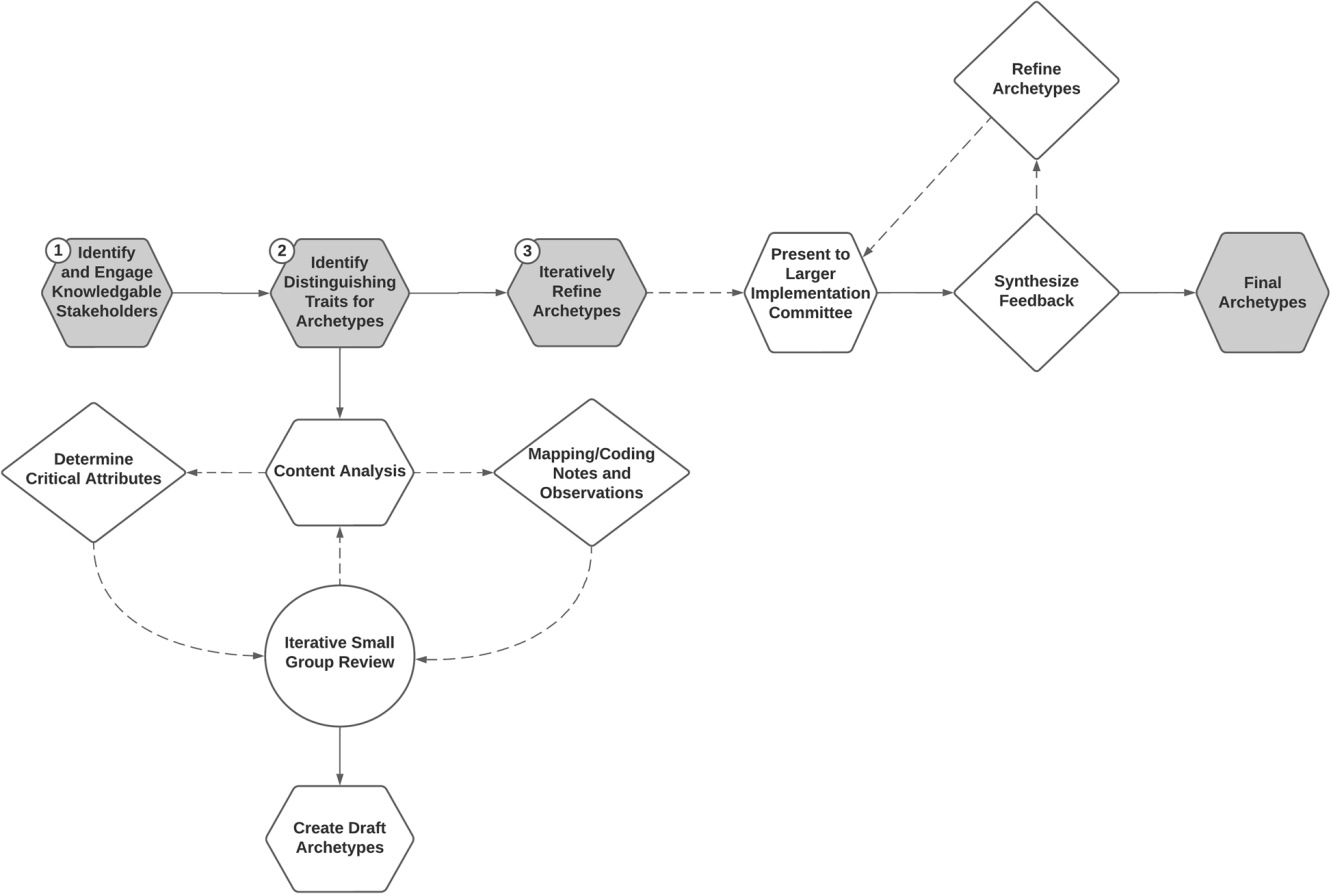
Process map for the development of archetypes

First, we provide an overview of the SHARING CHOICES trial, which serves as the case study for this methodology. Then, we describe our methodology for 1) selection of roles (i.e. implementation actors or targets) for archetype development and how the development and refinement of archetypes unfolded, 2) data collection and 3) archetype development. Figure 1 presents a process map summarizing the approach.

### SHARING Choices

SHARING Choices is a multicomponent advance care planning (ACP) communication intervention based in primary care. Components include: 1) outreach from the primary care practice introducing SHARING Choices to prepare patients and families to engage in ACP, 2) access to a facilitator trained in all elements of SHARING Choices, including Respecting Choices and ACP for persons with Alzheimer’s Disease and related dementias (ADRD) and their families, 3) person-family agenda-setting to align perspectives about the role of family and stimulate conversation about health care issues and ACP, 4) information about registration for the patient portal to enable and extend electronic interactions and information access to patients *and* family, and 5) ADRD educational materials and resources for staff and clinicians.

SHARING Choices was tested in a pragmatic trial within two health systems in the Baltimore/Washington DC region, with the goal of increasing ACP documentation and reducing potentially burdensome end of life care (NCT04819191). SHARING Choices implementation involved extensive shareholder engagement, pilot work and model development within partner systems; the study and intervention protocol have been detailed elsewhere [18, 19]. Intervention embedding occurred between September 2020 and March 2021 and SHARING Choices ACP conversations were conducted between February 2021 and April 2023. The decision to develop archetypes was motivated by human-centered design principles [6] and the need to improve our understanding of unexpected and unmeasured implementation processes related to both implementation actors and implementation targets. This is highly aligned with Normalization Process Theory, an implementation process model which helps implementation scientists focus on the range of people, situations and settings to make sense of how the change is being implemented by the people delivering and receiving the intervention [20]. Development of the archetypes began in February of 2021 in parallel with the initiation of the implementation of the SHARING Choices intervention in primary care clinics and after five months of experience embedding the intervention. Iterative development based on learnings from the ongoing implementation continued for archetypes as the trial progressed with final designs completed in May 2022.

### Step 1: Identify roles for archetype development

Identification of roles for archetype development should be based on relevance to the success of the specific intervention embedding and delivery. In the updated Consolidated Framework for Implementation Research, the implementation process domain suggests understanding both innovation deliverers (i.e. actors) and recipients (i.e. targets) [21]. For instance, implementation targets in the setting of healthcare interventions may be patients and families. For instance, clinics may be randomized in cluster pragmatic trials, so various implementation actors within clinics such as providers and staff could be appropriate for archetype development when it is important to understand their roles and interactions to improve implementation. In using implementation strategies such as champions or facilitators (i.e. implementation actors), archetypes are useful to plan or evaluate the implementation strategy or to train the actors using archetypes of implementation targets (i.e. patients).

#### Examples using SHARING Choices

We identified implementation actors within the SHARING Choices trial including the primary care champion and the ACP facilitator and we also identified implementation targets, primary care patients.

##### Primary care champion

The champion, broadly defined by Miech and colleagues is an individual who is internal to an organization with an “intrinsic interest and commitment to implementing a change.” This role has been referenced in implementation science literature for over 20 years [22]. Champions utilize inside knowledge to aid the adoption of interventions, particularly when designed by people external to the organization who do not have the same context [23]. Support from champions is an identified factor for implementation success [23–26]. In SHARING Choices, each clinic identified a primary care champion who was responsible for connecting research staff with clinic staff and leaders and guiding processes and workflows [18, 19]. This role was selected for archetype development based on our observation that champions were having variable success in integrating SHARING Choices into their primary care practices. We wanted to learn from these successes and challenges without directly identifying individuals.

##### Respecting Choices™ ACP facilitator

This role was first described in the 1990s [27, 28]; training to be a facilitator involves completing a manualized curriculum to lead ACP conversations that support but are adjacent to the patient’s healthcare team [29]. Over the past three decades, the Respecting Choices curriculum has been used to advance ACP across a range of health care settings [29]. This role was prioritized due to the importance of individual facilitator attributes. For example, a facilitator with high confidence in completing documentation but poor conversational skills may not engage patients or families well but produce complete ACP documentation. Conversely, a facilitator with strong conversational skills but poor documentation may have more conversations that are not recorded. By creating archetypes, we hoped to better understand how individual facilitator attributes may benefit from additional training and explain observed outcomes.

##### Primary care patient

Intended end users included new and established primary care patients ages ≥ 65 years with and without ADRD. Due to the diversity of patients that were targeted, we sought to identify relevant characteristics affecting receptivity to SHARING Choices to contextualize challenges and solutions available to facilitators in their interactions.

### Step 2: Identify shareholders and data sources for archetype development

Archetype development depends on identifying relevant shareholders and the optimal approach to eliciting their input in the development of archetypes for a given role (e.g., such as through informal interviews or shadowing individual or group interactions). Relevant shareholders may be individuals in the archetype role, those supervising or interacting with the archetype role professionally (i.e. managers) and those who receive services from individuals in the role (i.e. patients).

Data collection for the development of archetypes should reflect sources relevant to the implementation of the project goals, or in this case, the pragmatic trial. Generally, archetypes are intended to be used when detailed data are not available for users and not feasible to collect (i.e. in the midst of a trial). However, meta-data collected and documented in study team meetings such as notes taken during training sessions, sessions to support interventionists in a behavioral trial, notes from shareholder meetings (i.e. advisory boards), and even qualitative studies from preliminary work may be used to develop archetypes. Increasingly, study teams are working with patient and family advisory councils who may be well positioned to contribute to the development of archetypes. Research teams should consider if data collected requires ethical approval or if data collection for archetypes falls under the larger study IRB.

#### Examples using SHARING Choices

In the process of conducting the SHARING Choices trial we engaged different shareholders and had access to several sources of data to develop archetypes. To inform archetypes for the *champion* role, we drew on field notes from regular champion meetings which included 5 one-hour meetings and a bi-annual convening that included all champions from both health organizations. Topics for meetings included understanding clinic workflow, mapping activities to understand the involvement of the champion and discussion of the interactions between the clinic staff and the study team. To inform the archetypes for the *facilitator* role, we drew on field notes from 14 regular, bi-weekly facilitator meetings, led by ACP Facilitator Group Lead (VC). Weekly agendas focused on facilitator strengths and weaknesses, challenges, experiences with specific aspects of the intervention, interactions with champions, and embedding in clinic workflows (relationships with staff and logistical considerations). Team meetings were used to summarize meeting notes, review in-progress archetypes, and gather feedback. Field notes were taken during these meetings to further guide the development of relevant facilitator archetypes. Archetypes representing *patients* were developed with input from facilitators as approved IRB protocols did not allow for direct patient contact by the study team. We again drew on field notes from bi-weekly check-in meetings with facilitators. Facilitators shared patient attributes during discussions about the successes and challenges associated with the intervention delivery for the purpose of skills improvement.

### Step 3: Generate unique archetypes and their distinguishing traits

Before generating unique archetypes and distinguishing traits, the archetype team should first establish a shared understanding of the purpose of archetype development and refinement for the trial and intended future uses, making sure to emphasize the goal of understanding and optimizing project roles. Each archetype for a given role should have distinguishing traits such as prior experience, inhibitors, emotions, expectations, and what competencies are required for success in their role. These traits are common for archetype development but other traits may be appropriate [6, 7]. An appropriate analytic strategy should be used to construct archetypes based on available data. As archetypes evolve, attributes may be added or subtracted with additional layers of detail. At this stage it may be useful to combine or split archetypes to ensure that each is internally consistent and externally diverse. It is likely that several rounds of revisions will be necessary. When the team is satisfied that the archetypes represent important users and have sufficient detail, names of each archetype and full descriptions should be drafted prior to larger group review.

#### Examples using SHARING Choices

In SHARING Choices, the archetype development team reviewed their purpose, to capture key characteristics in a nonjudgmental way, recognizing that each archetype represented something unique and positive about the role in the study and the work. Content analysis of field notes and interviews was conducted by a human-centered design strategist (AJ) and a research assistant (AK). A small group (AJ, AK, DS) met regularly to discuss emerging content and interpretation of the data, emphasizing attributes and related emotions, to deepen our understanding and ameliorate the influence of our own biases [30]. For example, we recognized a type of advance care planning facilitator, which we referred to as the “Go-getter”. We leveraged our ACP facilitator training and meeting notes to qualitatively describe experiences, inhibitors, emotions, etc. We reviewed educational backgrounds of individuals in the role, assessed challenges they brought up during meetings and training needs for success in their role. Distinguishing traits for the Go-getter included strengths such as the ability to organize efficient workflows and initiative to solve problems. As we further developed other archetypes in this role, it was important to make sure that each archetype was distinct but did not represent any specific person who had worked in this role in our study. For instance, the Go-getter shared work productivity with another archetype we called the “Thorough”. The Thorough archetype consistently used existing workflows reliably and with excellence but did not exemplify flexibility or creativity as did the Go-getter. For each archetype, we developed a profile with identified strengths, challenges, prevailing emotions, and approaches for successful collaboration (“what works for me”).

### Step 4: Iteratively refine archetypes through exposure, scrutiny, and shareholder input

Draft archetypes for each role should be presented to a larger shareholder group. This may include shareholders such as study team leadership, collaborators, and organizational partners to strengthen clarity and usefulness. Several rounds of refinement will likely be needed between the larger and smaller groups before finalizing details of each archetype, supporting graphics and materials. Exemplar questions we posed to shareholders are listed in Table 1. As archetypes are used in practice such as hiring or training, it may be appropriate to engage a graphic designer to make the archetypes fit with organizational branding. Accompanying material such as instructions for use should be developed so that the archetypes can be used in the future.

**Table 1:** Questions for Shareholders

Refinement questions:	
Are these archetypes unique? Is there an important archetype that is missing?	
Do the attributes make sense and align with your experiences with the role (i.e. ACP facilitators)?	
Utility questions:	
What was the representation/number of users of each archetype in our trial or program?	
When thinking about a challenge encountered in the trial, how can these archetypes help us brainstorm solutions?	
How could these archetypes inform understanding of how our teams work(ed)?	
How can these archetypes help us interpret our results and implementation successes?	

#### Examples using SHARING Choices

In SHARING Choices, two larger groups reviewed and provided additional feedback. First, an implementation oversight committee, comprised of organizational partner leadership, clinicians, and study team members reviewed archetypes with the goal of ensuring representation and applicability across both sites. Second, the study Steering Committee met to review the draft archetypes with the goal of clarifying distinguishing attributes of archetypes for each role and ensuring that each archetype within a given role was sufficiently distinct. Finally, archetype names and descriptions were finalized. The study team worked closely with a graphic designer to create materials that could be used by future teams conducting ACP pragmatic trials with potential utility for other projects as well.

**Fig. 2:**
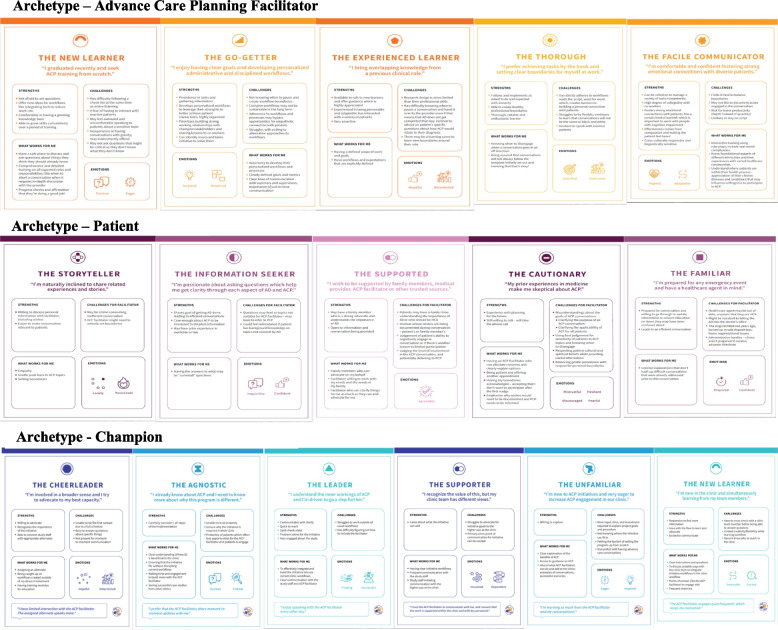
Archetypes for 3 Roles in SHARING Choices

Using this methodology, a total of 6 distinct archetypes for the champion role, 5 archetypes for the facilitator role and 6 archetypes for the patient role were developed. The names and descriptions of each archetype are presented by role in Table 2. All archetypes for each role are included in Fig. 2.

**Table 2:** Archetypes of Champions, Facilitators and Patients

Champions	
**Name of Archetype**	**Brief Description**
Cheerleader	Advocate to their best capacity and recognizes the importance of the initiative
New Learner	New in the clinic and is excited to communicate and learn from team members
Leader	Understands the inner workings of ACP and is willing to go a step further
Agnostic	Already aware of ACP and needs to know more about why this program is different
Supporter	Recognizes the value of the program, but the clinic team has different views
Unfamiliar	New to ACP initiatives and is eager to increase ACP engagement in their clinic
**Facilitators**	
Experienced Learner	Brings overlapping knowledge from previous clinical role and job in healthcare
Facile Communicator	Comfortable fostering strong emotional connections with diverse patient populations
New Learner	Seeks ACP training from scratch
Thorough	Prefers achieving tasks by the book and setting clear boundaries at work
Go-Getter	Enjoys clear goals and disciplined workflows
**Patients**	
Storyteller	Shares related experiences freely and readily opens up to facilitator
Information Seeker	Asks questions to understand details of ACP
Supported	Seeks support from family members who can advocate for the patient
Cautionary	Skeptical about the purpose and value of the intervention
Familiar	Already has an advance directive and is prepared to update it through an ACP conversation with the facilitator

## Discussion

We described our adaptation of a methodological approach to design archetypes for pragmatic trials in healthcare using SHARING Choices, a large pragmatic trial of an ACP intervention, as a case study example. We developed 17 archetypes representing three key implementation roles. The process of developing archetypes was useful to understanding our own team dynamics, personnel needs, support hiring and interpretation of process outcomes. The design and use of archetypes has potential application to increase partnership and support when embedding and scaling care delivery interventions but has not been well described to date in the peer review literature.

Human-centered design focuses on the needs and preferences of users to improve the user experience [6, 31]. The process of creating and using archetypes represents human-centered design in practice; bridging the knowledge throughout an organization with its designers who are developing a product for those individuals [6]. Archetypes have been used to inform patient and provider-facing educational materials and systems such as medication adherence programs, online patient portals, and patient support services, among others [8, 15]. They have also been used in the context of education initiatives and product design, such as developing patient-facing materials [32] and simulation-based training to foster person-centered care of health professionals [33]. In SHARING Choices, implementation actors (champions and facilitators) were critical to the successful delivery and adoption of SHARING Choices within two large, established community health systems in the Baltimore/Washington DC area. However, we collected minimal data about these actors. Archetypes were a way for us to summarize common actor experiences for group discussion, while recognizing future implementation trials should consider collecting data if feasible. Champions and facilitators are commonly used in scaling interventions, but we could not identify other ACP trials describing archetypes or personas of these critical roles.

We used reflexive monitoring of study team roles, an aspect of normalization process theory analysis and found archetypes particularly useful when evaluating team dynamics and hiring additional ACP facilitators [20]. In SHARING Choices, as facilitators changed throughout the trial, we saw a need to balance our team strengths. As an example, the Thorough archetype has strengths in executing tasks as dictated in a protocol; the team could be balanced with hiring someone who fits the Go-getter archetype which has strengths in building strong relationships and personalized workflows. The development and approach to archetype development led our team to consider new ways that archetypes *across* roles could collaborate and support one another, as well as archetypes that might be more challenged to collaborate. For instance, the Supporter, an archetype of the champion role and the Experienced Learner, an archetype of the facilitator role, may be a useful combination to achieve implementation goals. The Supporter recognizes the value of the program but may not excel at efficient patient communication concerning important care conversations. A facilitator who has a clear communication style would complement this individual and It may be valuable to look for characteristics of both archetypes when recruiting for these roles.

Due to their past use in product design and education, we envisioned our archetypes as tools with applications for better understanding implementation actor and targets and to aid in tailoring workflows and processes. It is important to note that we do not envision the use of archetypes as replacing shareholder engagement, but rather to enhance shareholder conversations about roles in an implementation trial and variation in experiences among implementation actors and targets. Archetype profiles can also be used to to shorten the gap between research and practice through iterative, person-centric activities [13, 34]. Health systems who adopt SHARING Choices may use our role definitions and archetypes to recruit, train and implement the program. As we continue to apply systems thinking to analysis of outcomes for SHARING Choices, we may need to further refine and adapt our archetypes to support patients and families and the champions and facilitators who support ACP [35].

### Limitations & strengths

Our approach to archetype development has several limitations. We did not plan to develop archetypes at the conception of the study. Therefore, it is likely that if we had planned earlier, more archetypes could have been developed and may have had a greater impact on our interactions with champions, facilitators and patients. Additionally, although our design process prioritized critical traits for each archetype, it is impossible to capture the full range of complex traits inherent to diverse groups. Moreover, people’s thoughts and behaviors are not static. For example, a patient could be considered a “Storyteller” at the start of their ACP journey, then experience memory decline and shift more toward the “Supported” archetype. Similarly, clinic champions could progress from the “New Learner” to the “Leader” through this process of change. This is potentially both a strength and a limitation. Also, we did not formally collect qualitative data to support the development of archetypes which would have been particularly informative for the patient archetypes. With more rigorous methods, more refined archetypes could be developed. Finally, it is difficult to quantify the value of using these archetypes to understand our experiences. Future studies may find innovative approaches to evaluate the value of archetypes.

Our work also has several strengths. First, we describe a methodology for the development of archetypes that we hope organizations will find useful when planning to implement new programs that involve multiple interacting roles. In SHARING Choices we actively engaged multiple levels of participants and partners and iteratively defined our archetypes. We used positive descriptions to reinforce the importance of not using archetypes to stereotype. Each time we shared the archetypes we emphasized that they did not represent individuals, but rather were used to help think through roles in SHARING Choices. The development of these archetypes strengthened our understanding of our personnel and future hiring needs and added a useful tool to the materials developed to support implementation.

## Conclusions

The design of archetypes is a useful strategy to contextualize understanding of the implementation process of a pragmatic trial. The methodology we describe includes analysis of common traits, needs, and emotions across the roles engaged in implementation. The development of archetypes improved our understanding of three critical roles in the SHARING Choices pragmatic implementation: patients, facilitators, and champions. *The archetypes and materials generated with them may be useful for future SHARING Choices and/or ACP implementation process improvement.* The process of developing these archetypes is tailored to this trial, however the development methodology has general implications for future implementation projects and pragmatic trials.

## Data Availability

The archetypes are made available in the supplementary materials. Data can be made available upon request to the corresponding author.
